# Heavy metal contamination in cosmetic hair dyes, dermal health risk assessment, and in vivo toxicological implications among exposed women in Khartoum, Sudan

**DOI:** 10.1016/j.toxrep.2026.102335

**Published:** 2026-08-26

**Authors:** Fatima Abdalaziz Hassan Mohamed, Badreddin M.H. Al-Hadiya, Raja Y. Alghadi, Ragaa Satti Mohmmed Abadi, Ahmed H. Bakheit

**Affiliations:** a Department of Pharmaceutics, Faculty of Pharmacy, Karary University, Khartoum, Sudan; b Department of Chemistry, Faculty of Science and Technology, Al-Neelain University, Khartoum, Sudan

**Keywords:** Cosmetic hair dyes, Heavy metals, Dermal risk assessment, EDI, Hazard quotient, Lifetime cancer risk

## Abstract

Cosmetic hair dyes can expose users to heavy metals, but integrated evidence combining product contamination, biomonitoring, and dermal risk assessment remains limited in Sudan. We quantified Cu, Cd, Hg, Pb, Mn, and Ni in eleven commercial black hair dyes using validated flame atomic absorption spectrometry. Blood samples from a pilot group of 19 women were analysed for selected metals, and a validated Arabic questionnaire assessed exposure practices and self-reported effects among 312 respondents. Consumer and occupational dermal risks were estimated using the estimated daily intake, hazard quotient, hazard index, and lifetime cancer risk. Manganese and lead were the predominant contaminants. Non-carcinogenic risk remained below the conventional threshold for individual products, but occupational exposure approached this threshold for high-lead products. Cadmium produced the clearest risk signal, with lifetime cancer risk above the commonly accepted range for occupational workers under the modelled assumptions. In the GFAAS pilot subset, detectable blood Pb occurred only among participants with at least 20 years of occupational exposure; this finding is exploratory and requires confirmation in a larger study. The results support mandatory pre-market metal screening, occupational biomonitoring, improved ventilation and personal protective equipment, and stronger surveillance of powdered and stone-based dyes in Sudan.

## Highlights

• First integrated Sudanese study on heavy metals in cosmetic hair dyes and exposed women.
• Eleven black hair dye products were analyzed for Cu, Cd, Hg, Pb, Mn, and Ni by validated AAS methods.
• Manganese and lead were the dominant contaminants, reaching 294.8 ppm and 29.5 ppm, respectively.
• Occupational users showed higher dermal risk, with cadmium presenting unacceptable lifetime cancer risk.
• Biomonitoring and questionnaire data support urgent cosmetic regulation and salon-worker protection in Sudan.

## Graphical Abstract

**Figure:**
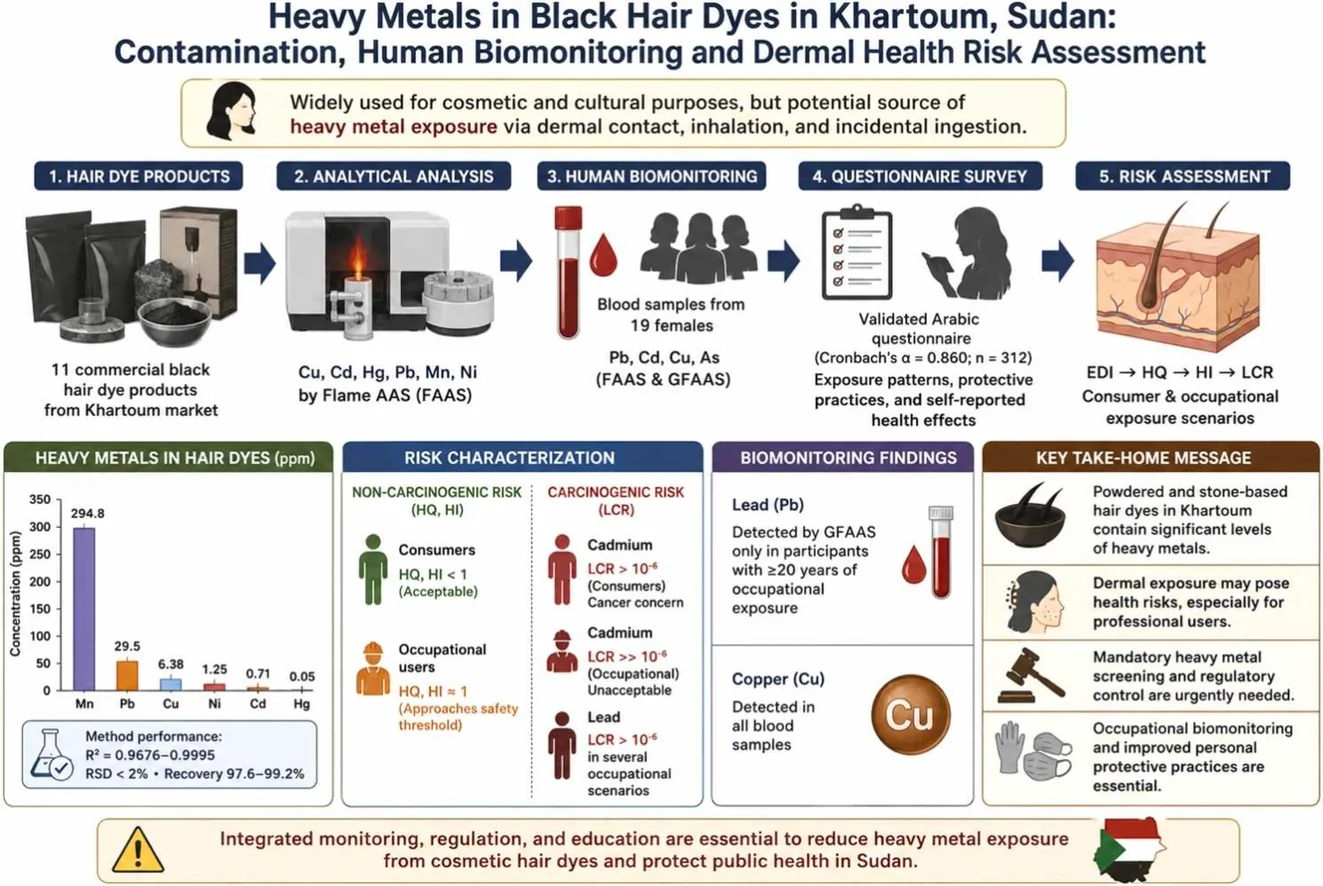

## 1 Introduction

Hair dyes are widely used worldwide and have important cultural and cosmetic roles in Sudan. Black powdered, stone-based, and cream formulations are applied to hair and, in some settings, to the skin. This pattern creates both consumer and occupational exposure, yet the toxicological burden of locally marketed products remains poorly characterized [1], [2].

Hair dye formulations are chemically complex, comprising oxidative intermediates such as para-phenylenediamine (PPD) and resorcinol, alkalising agents (ammonia, ethanolamine), hydrogen peroxide, and diverse adjuncts including surfactants, preservatives, and pigments [3]. Heavy metals may enter these formulations as residual impurities in raw materials (particularly mineral pigments), as contaminants from manufacturing equipment, or, in some regional markets, as deliberate colour-modifying additives such as lead acetate (a progressive blackening agent) [4]. The resulting metal burden can be substantial: documented concentrations in globally marketed products range from trace levels in GMP-compliant European formulations to hundreds of parts per million in products from poorly regulated markets [5], [6], [7].

The primary exposure pathway in hair dyeing is dermal contact with the scalp and periscalp skin—a vascularised, follicle-rich substrate with higher permeability than most body regions. Repeated contact enables transdermal metal absorption, systemic distribution, and organ accumulation [8]. Occupational beauty workers face additional exposure through inhalation of particulate dye aerosols during mixing and application, as well as incidental ingestion facilitated by the high frequency and volume of product handling [9]. Alarmingly, data from this study and the wider literature indicate that personal protective equipment (PPE) compliance among professional dye practitioners is low, substantially amplifying internal metal dose relative to consumers [10].

The toxicological consequences of the metals identified in this study are well established in the in vivo literature. Lead exerts neurotoxic, haematotoxic, and cardiovascular effects; manganese causes Parkinsonian-type neurodegeneration; cadmium is a Group 1 International Agency for Research on Cancer (IARC) carcinogen with nephrotoxic and endocrine-disrupting properties; mercury targets the central nervous system through thiol-group binding; and nickel is the most common occupational contact allergen and a recognised respiratory carcinogen [11], [12], [13], [14], [15]. Despite this evidence base, health risk assessments specifically calibrated to the dermal exposure scenario for hair dye users in low-income country markets remain rare, and none have been published for Sudan.

Integrated studies have remained scarce in Sudan because routine cosmetic surveillance, laboratory capacity for trace-metal analysis, and occupational biomonitoring programmes are limited, while human exposure data are rarely linked to product-specific measurements. The present study addresses this gap by: (i) quantifying six heavy metals in eleven commercially available black hair dyes by validated FAAS; (ii) conducting exploratory biomonitoring of Pb, Cd, Cu, and As in a pilot group of exposed Sudanese women; (iii) estimating consumer and occupational dermal risks using EDI, HQ, HI, and LCR; (iv) characterising plausible sources of metal contamination; and (v) interpreting the findings in relation to established in vivo toxicological evidence.

## 2 Materials and methods

### 2.1 Study design and ethics

A cross-sectional, institution- and community-based analytical study was conducted in Khartoum State, Sudan (2021–2022). Ethical clearance was obtained from the Khartoum State Ministry of Health Ethics Committee; written informed consent was obtained from all blood-sample donors and questionnaire respondents.

### 2.2 Hair dye sample collection

Eleven commercially available black hair dye products (coded HD-A through HD-L) representing powdered, stone-based, and cream formulations from seven countries (India, Indonesia, Egypt, Pakistan, China, France, and locally unbranded stone dyes) were purchased from Khartoum retail outlets between March and August 2021. Origin, formulation type, batch number, and expiry date were recorded for each product.

### 2.3 Blood sample collection and participant characteristics

A pilot biomonitoring group of 19 Sudanese women aged 19–56 years was purposively recruited to represent long-term occupationally exposed beauty workers and comparison participants with no current occupational hair-dye exposure. Eligibility required adulthood, residence in Khartoum State, willingness to provide a blood sample and exposure history, and absence of an acute illness at sampling. Occupational participants were selected to provide a range of exposure durations and product-handling frequencies. Because recruitment was non-probabilistic and the sample was small, the biomonitoring findings are exploratory and were not intended to provide population prevalence estimates. Venous blood (∼5 mL) was collected into EDTA-anticoagulant tubes and refrigerated (≤4 °C) before analysis. Ten plasma samples were processed by APDC/MIBK extraction for FAAS, while nine whole-blood samples were wet-acid digested (HNO₃/H₂O₂) for GFAAS.

### 2.4 Questionnaire survey

A structured, interviewer-administered, 42-item Arabic-language questionnaire was used. It covered five domains: sociodemographic characteristics; personal hair-dye use; occupational exposure; protective practices; and self-reported symptoms during or after product handling. Content validity was assessed before field use, and internal consistency was acceptable (Cronbach's alpha = 0.860). The minimum sample size was calculated as 300 using Z = 1.96, an expected proportion of 0.50, a precision of 0.07, and a design effect of 1.5.

The questionnaire was administered to 312 women selected through multistage cluster sampling across three Khartoum localities. Responses were coded and entered into SPSS Version 28. Categorical items were summarized using frequencies and percentages. Reported symptoms were treated as self-reported outcomes and were not interpreted as physician-confirmed diagnoses. The final sample exceeded the calculated minimum sample size of 300 participants.

The questionnaire was translated into Arabic and independently back-translated into the source language. The original and back-translated versions were compared to assess conceptual, linguistic, and cultural equivalence, and discrepancies were resolved before field administration.

### 2.5 Sample preparation

For determination of Cu, Cd, Pb, Mn, and Ni, approximately 5.0 g of each product was digested with 40 mL of freshly prepared HCl:HNO_3_ (3:1, v/v; 30 mL HCl and 10 mL HNO3) on a sand bath at 220 ° C for 30 min. After cooling, each digest was diluted to 200 mL with deionised water and filtered through Whatman No. 42 filter paper. Mercury was determined from a separate aliquot digested with HNO_3_:HClO_4_ (1:1, v/v) and concentrated H_2_SO_4_ at 220 ° C. Reagent blanks were processed with each digestion batch. Sulfuric acid was restricted to the mercury-specific preparation and was not used for routine multi-element digestion.

### 2.6 Instrumentation and method validation

Flame Atomic Absorption Spectrometry (FAAS) measurements used a Varian 220 instrument at characteristic wavelengths (Pb: 217.0 nm; Cd: 228.8 nm; Hg: 253.7 nm; Cu: 324.8 nm; Mn: 279.5 nm; Ni: 232.0 nm). Graphite Furnace Atomic Absorption Spectrometry (GFAAS) measurements (Pb, Cd, Cu, As) used a Varian 7000. Validation parameters—linearity (R²), limit of detection (LOD), limit of quantification (LOQ), precision relative standard deviation (RSD%), and accuracy (recovery%)—were determined as described in 3.1.

### 2.7 Dermal health risk assessment framework

Quantitative dermal risk assessment followed USEPA guidance and published cosmetic-risk approaches using consumer and occupational scenarios. Metal-specific dermal absorption fractions were selected from published experimental or regulatory sources and were applied consistently across products: Cu 0.10, Cd 0.05, Hg 0.03, Pb 0.03, Mn 0.03, and Ni 0.06. These values represent screening-level estimates rather than formulation-specific measurements. Because absorption may vary with metal speciation, vehicle, skin condition, contact time, and occlusion, the resulting HQ, HI, and LCR values should be interpreted as model-based estimates rather than direct measures of absorbed dose [10], [16], [17], [18]

#### 2.7.1 Estimated Daily Intake (EDI)

The dermal EDI (mg kg⁻¹ day⁻¹) was calculated as:

(1) $$\mathrm{EDI}=\;\frac{C\times \mathrm{DE}\times \mathrm{DAF}}{\mathrm{BW}\times 1000}$$

where C is the metal concentration in the hair dye product (mg kg⁻¹ ≡ ppm); daily exposure equivalent (DE) is the daily dermal exposure equivalent (g day⁻¹) computed as (amount per application × application frequency)/365; the dermal absorption fraction (DAF) is the metal-specific dermal absorption fraction (dimensionless); and body weight (BW) is body weight (kg). DE for consumers was 1.644 g day⁻¹ (50 g/use × 12 uses/year ÷ 365); for occupational workers, 142.47 g day⁻¹ (200 g day⁻¹ × 260 working days ÷ 365 days). BW = 60 kg was the mean body weight determined from the study participants.

#### 2.7.2 Hazard Quotient (HQ) and Hazard Index (HI)

Non-carcinogenic risk was assessed as:

(2) $$\mathrm{HQ}=\frac{\mathrm{EDI}}{\mathrm{RfD}}$$

and:

(3) $$\mathrm{HI}=\sum HQ_{i}$$

where the reference dose (RfD) is the USEPA oral reference dose (mg kg⁻¹ day⁻¹) used as a surrogate chronic dermal reference value [18]. A compound with HQ < 1 poses acceptable non-carcinogenic risk; HI ≥ 1 for the mixture indicates likely adverse effects [18].

#### 2.7.3 Lifetime Cancer Risk (LCR)

For metals with established carcinogenic slope factors (CSF), LCR was estimated as:

(4) $$\mathrm{LCR}={\mathrm{CDL}}_{\mathrm{nl}}^{\mathrm{festmdest}}\times \mathrm{CSF}$$

where

(5) $${\mathrm{CDL}}_{\mathrm{nl}}^{\mathrm{festmdest}}=\frac{\mathrm{EDI}\times (\mathrm{ED}\times 365)}{\mathrm{ATc}}\;$$

where ATᴄ = 70 × 365 = 25,550 days (carcinogenic averaging time); exposure duration (ED) is exposure duration (years); and CSF is the oral cancer slope factor (mg kg⁻¹ day⁻¹)⁻¹ . CSF values applied were 0.0085 (mg kg⁻¹ day⁻¹)⁻¹ for Pb, Office of Environmental Health Hazard Assessment (OEHHA) and 15.1 (mg kg⁻¹ day⁻¹)⁻¹ for Cd (OEHHA oral), reflecting IARC Group 2B and Group 1 classifications, respectively. An LCR < 10⁻⁶ is generally considered negligible; 10⁻⁶ ≤ LCR ≤ 10⁻⁴ is tolerable and requires management; LCR > 10⁻⁴ is unacceptable [18]. All parameters are summarised in Table 5.

### 2.8 Statistical analysis

Descriptive statistics were computed in SPSS v.28. Questionnaire data were summarised using frequencies and percentages. Inferential associations between symptoms and dye formulation or exposure duration were not estimated because product type and symptoms were self-reported, participants could use multiple formulations, and the survey was not designed to establish temporality or product-specific causation. This analytical limitation is now stated explicitly. The exposure–Pb relationship was evaluated graphically in the pilot biomonitoring group. Risk quotients are presented as point estimates, and uncertainty is discussed qualitatively.

## 3 Results

### 3.1 Method validation

All six calibration curves showed excellent linearity (R² = 0.9676–0.9995; Table 1). LOD ranged from 0.125 mg/L (Cd) to 4.01 mg/L (Ni); LOQ from 0.378 to 12.16 mg/L. Precision (RSD < 2%) and recovery (97.6–99.2%) confirmed method suitability for trace metal determination in complex cosmetic matrices.

**Table 1:** Analytical validation parameters for FAAS determination of Cd, Cu, Hg, Pb, Mn, and Ni in hair dye matrices.

**Metal**	**λ (nm)**	**Linear range (mg/L)**	**Regression equation**	**R²**	**LOD (mg/L)**	**LOQ (mg/L)**	**RSD (%)**	**Recovery (%)**
Cd	228.8	0.5–3	A = 0.02124 C − 0.00017	0.9989	0.125	0.378	1.4	98.7
Cu	324.8	5–20	A = 0.00617 C + 0.00045	0.9976	1.11	3.38	1.6	99.2
Hg	253.7	10–60	A = 0.00816 C − 0.00142	0.9991	2.39	7.24	1.8	97.9
Pb	217.0	5–20	A = 0.00166 C − 0.00008	0.9995	0.48	1.46	1.5	98.5
Mn	279.5	5–20	A = 0.00435 C + 0.00045	0.9968	1.20	3.64	1.7	99.0
Ni	232.0	5–20	A = 0.00503 C + 0.00177	0.9676	4.01	12.16	1.9	97.6

### 3.2 Heavy metal concentrations in hair dye products

Metal concentrations in the eleven products are presented in Table 2. Manganese was the most abundant contaminant, with HD-J (Indian powder) registering 294.8 ppm, HD-C (Indian powder) 163.4 ppm, and HD-I (Indian powder) 155.2 ppm. Lead was detected in three products (HD-E: 29.5 ppm; HD-J: 17.5 ppm; HD-L: 7.99 ppm), all exceeding the EU technically-avoidable limit of 2 ppm [19]. Mercury was present in HD-H (0.929 ppm), HD-E (0.136 ppm), and HD-A (0.008 ppm). Cadmium was found in HD-D (0.853 ppm) and HD-L (0.123 ppm). Nickel was detected in HD-C (6.10 ppm), HD-B (2.30 ppm), HD-A (1.70 ppm), and HD-J (0.79 ppm). The two European-regulated cream products (HD-F, HD-G) contained no detectable metals, contrasting sharply with Asian-origin powdered formulations.

**Table 2:** Heavy metal concentrations (ppm) in eleven commercial hair dye products (FAAS).ND= not detected.

**Sample**	**Formulation**	**Origin**	**Cu (ppm)**	**Cd (ppm)**	**Hg (ppm)**	**Pb (ppm)**	**Mn (ppm)**	**Ni (ppm)**
HD-A	Powder	Indonesia	nd	nd	0.008	nd	nd	1.699
HD-B	Powder	India	nd	nd	0.026	nd	19.43	2.299
HD-C	Powder	India	4.721	nd	nd	2.510	163.41	6.096
HD-D	Stone	—	nd	0.853	nd	0.786	41.78	nd
HD-E	Powder	—	nd	nd	0.136	29.53	nd	nd
HD-F	Cream	France	nd	nd	nd	nd	nd	nd
HD-G	Cream	—	nd	nd	nd	nd	nd	nd
HD-H	Cream	Egypt	nd	nd	0.929	nd	13.63	nd
HD-I	Powder	India	nd	nd	nd	nd	155.25	nd
HD-J	Powder	India	3.096	nd	nd	17.48	294.80	0.791
HD-K	Powder	China	nd	nd	nd	2.886	18.18	nd
HD-L	Cream	Pakistan	nd	0.123	nd	7.993	6.409	nd

### 3.3 Blood lead levels by FAAS

All ten FAAS plasma samples contained measurable Pb (Table 3), ranging from 0.51 to 0.88 ppm (mean±SD: 0.673 ± 0.130 ppm). Even the control participant registered 0.52 ppm, indicating background environmental Pb exposure. The highest Pb levels (0.82 and 0.88 ppm) occurred in participants with 22 and 10 years of occupational exposure accompanied by high-volume usage.

**Table 3:** Blood Pb concentrations (FAAS) with participant exposure profiles and clinical notes.

**Sample**	**Age (yr)**	**Blood Pb (ppm)**	**Status**	**Disease**	**Dye type**	**Exp. (yr)**	**Usage**	**Reported symptoms**
1 F	40	0.82	Married	Hypertension	Henna-A	22	3 btl/wk	Headache; hypertension
2 F	56	0.78	Married	None	D-7yr, Henna-A	25	12 btl/wk	Itching; sinusitis, allergy
3 F	33	0.53	Married	None	D-2yr, Henna-A	12	3 btl/wk	Throat pain
4 F	25	0.69	Single	None	Henna-A	5	6 btl/wk	None
5 F	32	0.65	Married	Thyroidism	D-10yr, Henna-A-15yr	25	2 kg D/mo	Allergy; ↑prolactin
6 F	38	0.51	Married	None	Henna-A	12	1 btl/wk	Flu exacerbation
7 F	50	0.88	Married	(HTN) + DM	Henna-A	10	2 btl/wk	None
8 F	25	0.52	Married	None	Henna-A	5	Control	Allergy
9 F	47	0.72	Married	None	D-4yr, Henna-A	28	10 btl/wk	Allergy
10 F	19	0.63	Single	None	Henna-A	4	4 btl/wk	Eye allergy

*diabetes mellitus (DM), Hypertension (HTN)

### 3.4 Blood heavy metals by GFAAS

Among nine whole-blood samples analyzed by GFAAS (Table 4), cadmium and arsenic were non-detectable in all cases. Lead was detectable in three samples corresponding exclusively to participants with ≥ 20 years of occupational exposure: BS-7GF (0.015 ppb; 20 yr), BS-8GF (0.036 ppb; 21 yr), and BS-9GF (0.058 ppb; 37 yr). The highest Pb level (BS-9GF, 37-year practitioner handling 15 bottles/week) co-occurred with the most severe symptom profile: sinusitis, blurred vision, eye redness, hypertension, and hypercholesterolaemia. Copper was ubiquitously detectable (0.115–0.860 ppb; mean 0.358 ± 0.319 ppb) with high inter-individual variability reflecting combined dietary, metabolic, and occupational contributions.

**Table 4:** Blood heavy metal concentrations (ppb) by GFAAS.ND= not detected; © = control.

**Sample**	**Age (yr)**	**Pb (ppb)**	**Cu (ppb)**	**Status**	**Disease**	**Exp. (yr)**	**Usage**	**Reported symptoms**
1GF©	37	nd	0.119	M	None	12	Control	None
2GF©	40	nd	0.730	M	None	12	Control	None
3GF©	37	nd	0.115	S	HTN + Thyroid.	10	Control	None
4GF©	21	nd	0.740	S	None	4	Control	None
5GF	27	nd	0.136	M	None	12	6 btl/wk	Black mucus
6GF	35	nd	0.860	S	Sinusitis	17	4 btl/wk	Nasal itch; sinusitis
7GF	37	0.015	0.128	M	None	20	20 btl/wk	Tearing; blurred vision
8GF	48	0.036	0.136	M	None	21	5 btl/wk	Sinusitis
9GF	50	0.058	0.260	M	HTN + ↑Chol.	37	15 btl/wk	Sinusitis; blurred vision; eye redness; HTN

* bottles per week (btl/wk),

### 3.5 Dermal health risk assessment

Exposure parameters and toxicological reference values used for risk calculations are detailed in Table 5&6.

**Table 5:** Risk assessment exposure parameters and toxicological reference values. DAF = dermal absorption fraction; RfD = reference dose; CSF = cancer slope factor; AT_C = carcinogenic averaging time; BW = body weight.

**Parameter**	**Symbol**	**Consumer**	**Occupational**	**Unit / Source**
Amount per application	m	50	200	g; Scientific Committee on Consumer Safety (SCCS) default / this study
Application frequency	F	12 (1/month)	260 (working days)	times yr⁻¹
Daily exposure equivalent	DE	1.644	142.47	g day⁻¹ ; calculated
Body weight	BW	60	60	kg; this study (mean female weight)
Exposure duration	ED	25	20	years; this study participant range
Non-carcinogenic AT	ATᴄ	9125	7300	days (ED×365); USEPA
Carcinogenic AT	ATᴄ	25,550	25,550	days (70 yr×365); USEPA

**Table 6:** Key dermal exposure parameters (DAF, RfD, IARC classification, CSF) for selected metals from USEPA and supporting sources; oral RfDs used as dermal surrogates, with no established dermal CSF for nickel.

**Metal**	**DAF**	**RfD (mg kg⁻¹ day⁻¹)**	**IARC Classification**	**CSF (mg kg⁻¹ day⁻¹)⁻¹**	**Reference**
Cu	0.10	4.0 × 10⁻²	Group 3	N/A	USEPA, Integrated Risk Information System (IRIS); SCCS
Cd	0.05	1.0 × 10⁻³	Group 1	15.1	USEPA IRIS; OEHHA oral
Hg (inorg.)	0.03	3.0 × 10⁻⁴	Group 3	N/A	USEPA IRIS
Pb	0.03	3.5 × 10⁻³	Group 2B	8.5 × 10⁻³	USEPA IRIS; OEHHA oral
Mn	0.03	1.4 × 10⁻¹	Group 3	N/A	USEPA IRIS
Ni	0.06	2.0 × 10⁻²	Group 1*	N/A*	USEPA IRIS; *dermal CSF unestablished

DAF sources: Pb, Hg, Mn — USEPA [20]; Cu — SCCS/1611/19; Cd — Hostynek [21]; Ni — Filon et al# (2009). RfD = oral reference dose used as chronic dermal surrogate per USEPA [20]. *Ni IARC Group 1 based on inhalation; dermal cancer slope factor not formally established.

Sensitivity of the screening estimates to DAF assumptions was evaluated proportionally. Because EDI, HQ, and LCR are linear functions of DAF, a 50% lower DAF produces values 50% below the reported estimates, whereas a twofold higher DAF doubles them. This range does not replace formulation-specific permeability testing, but it shows which conclusions are robust to plausible uncertainty. The occupational Cd signal for HD-D remains above 10⁻⁴ at the reported DAF and remains above 10⁻⁶ even if the assumed DAF is reduced tenfold; other borderline estimates should be interpreted more cautiously.

### 3.6 Non-carcinogenic risk: Hazard Quotients (HQ) and Hazard Index (HI)

Table 7 presents HQ values for each detected metal and the composite HI for the consumer scenario. All individual HQ values were well below 1.0, and HI remained below 1.0 in all products, with the highest value observed for HD-E (HI = 7.31 ×10⁻³), driven almost exclusively by Pb. This indicates that non-carcinogenic risk from personal hair dye use is broadly acceptable under the modelled conditions.

**Table 7:** Hazard Quotient (HQ) and Hazard Index (HI) for the consumer exposure scenario (personal use, 50 g/application, 12 ×/year, ED=25 yr).ND= metal not detected.

**Sample**	**HQ(Cu)**	**HQ(Cd)**	**HQ(Hg)**	**HQ(Pb)**	**HQ(Mn)**	**HQ(Ni)**	**HI**
HD-A	ND	ND	1.90 × 10⁻³	ND	ND	1.21 × 10⁻²	**1.40 × 10⁻²**
HD-B	ND	ND	6.06 × 10⁻³	ND	9.89 × 10⁻³	1.64 × 10⁻²	**3.23 × 10⁻²**
HD-C	2.80 × 10⁻²	ND	ND	5.11 × 10⁻²	8.31 × 10⁻²	4.34 × 10⁻²	0.206
HD-D	ND	1.01 × 10⁻¹	ND	1.60 × 10⁻²	2.13 × 10⁻²	ND	0.139
HD-E	ND	ND	3.23 × 10⁻²	0.601	ND	ND	**0.633**
HD-F	ND	ND	ND	ND	ND	ND	ND
HD-G	ND	ND	ND	ND	ND	ND	ND
HD-H	ND	ND	0.221	ND	6.94 × 10⁻³	ND	0.228
HD-I	ND	ND	ND	ND	7.90 × 10⁻²	ND	**7.90 × 10⁻²**
HD-J	1.84 × 10⁻²	ND	ND	0.356	0.150	5.63 × 10⁻³	**0.530**
HD-K	ND	ND	ND	5.87 × 10⁻²	9.25 × 10⁻³	ND	**6.80 × 10⁻²**
HD-L	ND	1.46 × 10⁻²	ND	0.163	3.26 × 10⁻³	ND	0.180

Bold HI values ≥ 0.5 indicate elevated concern, particularly if exposure to multiple products is concurrent. Occupational HI for HD-E and HD-J approaches the acceptable threshold of 1.0.

Under the occupational exposure scenario (Table 8), HQ values for several metals increased ∼87-fold owing to the higher daily dermal contact. While HI remained below 1.0 for all products, two products exhibited HI values approaching the threshold: HD-E (HI = 0.633; Pb dominant) and HD-J (HI = 0.530; combined Pb + Mn). Products HD-C (HI = 0.206), HD-H (HI = 0.228), and HD-D (HI = 0.139) also showed elevated occupational HI values. When occupational exposure to multiple products simultaneously is considered, cumulative HI could plausibly exceed 1.0.

**Table 8:** Lifetime Cancer Risk (LCR) for Pb and Cd in products with detectable concentrations. Acceptable: LCR < 10⁻⁶; Tolerable: 10⁻⁶ ≤ LCR ≤ 10⁻⁴; Unacceptable: LCR > 10⁻⁴.

**Sample**	**Metal**	**C (ppm)**	**CSF (mg/kg/d)⁻¹**	**LCR Consumer**	**LCR Occupational**	**Risk Level (Occupational)**
HD-C	Pb	2.510	8.5 × 10⁻³	6.26 × 10⁻⁹	4.34 × 10⁻⁷	Acceptable
HD-D	Cd	0.853	15.1	6.30 × 10⁻⁶†	4.37 × 10⁻⁴††	Unacceptable
HD-D	Pb	0.786	8.5 × 10⁻³	1.96 × 10⁻⁹	1.36 × 10⁻⁷	Acceptable
HD-E	Pb	29.53	8.5 × 10⁻³	7.37 × 10⁻⁸	5.11 × 10⁻⁶†	Tolerable
HD-J	Pb	17.48	8.5 × 10⁻³	4.36 × 10⁻⁸	3.02 × 10⁻⁶†	Tolerable
HD-K	Pb	2.886	8.5 × 10⁻³	7.20 × 10⁻⁹	4.99 × 10⁻⁷	Acceptable
HD-L	Cd	0.123	15.1	9.09 × 10⁻⁷	6.30 × 10⁻⁵	Tolerable
HD-L	Pb	7.993	8.5 × 10⁻³	1.99 × 10⁻⁸	1.38 × 10⁻⁶†	Tolerable

† LCR > 10⁻⁶ (exceeds acceptable threshold; requires risk management). †† LCR > 10⁻⁴ (unacceptable; immediate action required). Consumer: ED= 25 yr; Occupational: ED= 20 yr; ATᴄ= 25,550 days. CSF: Pb = OEHHA oral; Cd = OEHHA oral. Cd LCR should be interpreted cautiously as oral CSF may overestimate dermal carcinogenic potency; nonetheless the signal is clear across all reasonable parameter sets.

### 3.7 Carcinogenic risk: Lifetime Cancer Risk (LCR)

Carcinogenic LCR was calculated for Pb (IARC Group 2B) and Cd (IARC Group 1) in products where these metals were detected (Table 8). Under the consumer scenario, all LCR values from Pb were below the 10⁻⁶ acceptable threshold (range 6.26 ×10⁻⁹ to 7.37 ×10⁻⁸). However, the Cd LCR from HD-D was 6.30 × 10⁻⁶, exceeding the 10⁻⁶ threshold even for personal use, placing this product in the ‘tolerable’ risk category requiring risk management intervention.

Under the occupational scenario, the risk picture changed markedly. Pb LCR exceeded 10⁻⁶ in three products: HD-E (5.11 ×10⁻⁶), HD-J (3.02 ×10⁻⁶), and HD-L (1.38 ×10⁻⁶)—all in the tolerable zone. Most critically, Cd LCR in HD-D for occupational workers reached 4.37 × 10⁻⁴, exceeding the 10⁻⁴ upper-bound threshold and entering the unacceptable risk zone, demanding immediate regulatory and engineering controls. The combined Pb + Cd LCR for HD-D occupational workers (4.37 ×10⁻⁴) constitutes the primary risk signal of this study.

### 3.8 Exposure duration–blood lead relationship and questionnaire findings

GFAAS data confirmed a threshold-type exposure–response pattern: Pb was undetectable in participants with ≤ 17 years of occupational exposure and emerged at increasing concentrations in those with 20, 21, and 37 years, consistent with progressive skeletal Pb accumulation and re-mobilisation. FAAS data showed a general positive association between exposure duration and blood Pb. The questionnaire (n = 312) found that 47.6% of women used hair dye; 84.7% applied dye to skin. Only 60% of occupational users reported consistent glove use; 44% used face masks. Symptom prevalence included hair loss (16.9%), headache (7.0%), and scalp irritation (3.8%); occupational users additionally reported sinus irritation (12%), nail changes (24%), and neurological-type symptoms (see 4.3).

## 4 Discussion

### 4.1 Sources and mechanisms of heavy metal contamination in hair dyes

The observed metal profiles differed by formulation and product origin. Powdered products, particularly HD-C, HD-I, and HD-J, contained the highest manganese concentrations, whereas the tested cream products generally contained lower concentrations. Because only eleven products were assessed and brands were anonymised, these differences should not be interpreted as representative of all products from a country or formulation category. They nevertheless indicate that raw-material composition, mineral pigments, manufacturing conditions, and quality control may contribute to product-to-product variability.

The high manganese concentrations in several powdered dyes may reflect manganese-containing mineral pigments or contamination of mineral raw materials [22]. However, metal speciation and pigment composition were not measured; deliberate addition cannot therefore be concluded from total manganese concentration alone. Similarly, the metals detected in the stone-based product may originate from geological raw materials, but this explanation requires mineralogical confirmation.

Lead detected in HD-E, HD-J, and HD-L may have originated from contaminated mineral pigments, raw materials, processing equipment, or, less likely, lead-containing colour modifiers. The present analytical method quantified total lead and cannot distinguish among these sources or chemical forms. Accordingly, source attribution remains hypothetical and should be tested through product-composition records, speciation analysis, and manufacturing audits.

Mercury at sub-ppm concentrations (HD-A, HD-E, HD-H) likely originates from impurities in preservative systems, particularly phenylmercuric compounds formerly common in cosmetic preservation. Although most regulatory frameworks have restricted Hg to near-zero, unregistered or poorly quality-controlled products may contain residual Hg from non-compliant preservatives [23]. Cadmium and nickel at trace levels are consistent with contamination from zinc oxide or titanium dioxide pigments that carry Cd as an ore-associated impurity, and from stainless-steel processing equipment that releases Ni and Cr during manufacturing [24]. The complete absence of detectable metals in European products (HD-F, HD-G) confirms that compliance with GMP and rigorous raw material specification testing effectively eliminates heavy metal contamination, demonstrating the technical feasibility of clean formulation.

Within the formulation matrix, heavy metal speciation is altered by the strongly alkaline and oxidising conditions of hair dye application (pH 9.5–10.5; H₂O₂ present). Lead(II) species are oxidised to Pb(IV) or form insoluble Pb(OH)₂ precipitates, which may be less dermally bioavailable than ionic Pb²⁺. Conversely, Cd²⁺ and Ni²⁺ remain predominantly as free ions under these conditions, enhancing their skin-penetrating potential [8]. The complexation of metals by PPD oxidation products and melanin precursors may also modulate bioavailability in a metal- and formulation-specific manner, introducing uncertainty into exposure assessments based solely on total metal content.

### 4.2 Interpretation of non-carcinogenic risk (HQ and HI)

The finding that HI < 1 across all products and both exposure scenarios (at the individual-product level) is reassuring for infrequent personal use but must be interpreted with caution. The HI framework conventionally assumes exposure to a single product, whereas many consumers and practically all beauty workers are exposed to multiple products concurrently or sequentially. In the occupational setting, the combined HI across even two high-burden products (e.g., HD-E plus HD-J) would approach or exceed 1.0, particularly through multiple routes (dermal + inhalation).

The Pb HQ for occupational workers using HD-E reached 0.601—the single largest contributor to risk across all metals and scenarios. This near-threshold value is driven by the combination of HD-E’s high Pb content (29.5 ppm), the low Pb RfD (3.5 ×10⁻³ mg kg⁻¹ day⁻¹), and the high occupational daily exposure. The Pb RfD has no threshold (WHO and USEPA acknowledge the absence of a safe exposure level for Pb), suggesting that even HQ values substantially below 1.0 warrant precautionary concern.

Mercury in HD-H (0.929 ppm) generated an occupational HQ of 0.221—the second-highest single-metal value observed. Despite being below the 1.0 threshold, this value is notable because the Hg RfD (3.0 ×10⁻⁴ mg kg⁻¹ day⁻¹) represents a conservative threshold for daily inorganic Hg exposure, and inorganic Hg in the gut can be partially methylated to neurotoxic MeHg by intestinal microbiota, effectively increasing the toxicological potency of ingested Hg. The combined HI for HD-H occupational workers (0.228) from Hg + Mn captures only dermal pathways; inclusion of inhalation would increase this estimate substantially.

### 4.3 Carcinogenic risk characterisation

The most critical finding of the risk assessment is the unacceptable carcinogenic risk from dermal Cd exposure for occupational workers using HD-D (LCR = 4.37 ×10⁻⁴). This value exceeds the 10⁻⁴ upper-bound threshold—the concentration above which the USEPA deems risk unacceptable regardless of socioeconomic considerations [18]. Although this calculation uses the OEHHA oral CSF for Cd (15.1 (mg kg⁻¹ day⁻¹)⁻¹ ), which may overestimate dermal risk due to lower dermal-to-oral bioavailability for Cd (estimated factor of 5–10 × difference [25]), even a tenfold correction yields an LCR of 4.37 × 10⁻⁵—still above the 10⁻⁶ threshold. This finding is consistent with the increasing recognition of dermal Cd exposure as a non-trivial carcinogenic pathway in occupational settings [25].

For Pb, the occupational LCR from HD-E (5.11 ×10⁻⁶) and HD-J (3.02 ×10⁻⁶) both exceed 10⁻⁶ but remain below 10⁻⁴, placing them in the tolerable zone. However, the OEHHA Pb CSF (8.5 ×10⁻³) is itself derived from epidemiological studies in adults, and recent evidence suggests that Pb carcinogenicity extends to lung, kidney, and stomach cancers at exposures below what was previously considered threshold, implying that the current CSF may underestimate true risk [26].

Notably, the Cd carcinogenic risk to consumers using HD-D (LCR = 6.30 ×10⁻⁶) marginally exceeded the 10⁻⁶ acceptable threshold, signalling that even occasional personal use of Cd-contaminated products carries a small but non-negligible excess cancer probability over a lifetime. HD-L, with both Cd (0.123 ppm) and Pb (7.99 ppm), presented combined occupational LCR of 6.44 × 10⁻⁵—in the tolerable zone but worth monitoring given that the product also contains measurable Mn as a non-carcinogenic neurotoxin.

### 4.4 In Vivo toxicological effects of the detected heavy metals

The toxicological effects of heavy metals depend on their chemical form, bioavailability, absorbed dose, exposure route and duration, and individual susceptibility. Repeated exposure may lead to tissue accumulation and adverse effects on the nervous, renal, cardiovascular, and haematopoietic systems. Lead disrupts neuronal signalling and haem synthesis and may contribute to neurotoxicity, anaemia, renal injury, and cardiovascular dysfunction. Excessive manganese exposure, particularly through inhalation of powdered products, can cause oxidative stress, mitochondrial dysfunction, neuroinflammation, and motor disturbances. Cadmium accumulates mainly in the kidneys, causes renal tubular and bone damage, impairs DNA repair, and has carcinogenic potential. Mercury disrupts antioxidant enzymes and mitochondrial function, with the kidneys and nervous system as major targets; however, the toxicity of mercury varies considerably among its chemical forms and exposure routes. Nickel is primarily relevant to dermal exposure as a sensitizer that may cause allergic contact dermatitis, whereas its strongest carcinogenic evidence is associated with occupational inhalation. Although copper is essential, excessive bioavailable copper may promote oxidative stress and cellular injury. These mechanisms provide biological plausibility but do not establish that the self-reported symptoms in this cross-sectional study were caused by metal exposure, particularly given the small biomonitoring sample, potential co-exposure to organic hair-dye ingredients, and unmeasured background sources of metals. The dietary findings of [27] support the cumulative toxicity of Pb, Cd, and Hg, but their ingestion-specific assumptions should not be applied directly to dermal exposure.

#### 4.4.1 Lead (Pb)

Lead exerts toxicity through multiple molecular mechanisms operating simultaneously across target organs. The core event is competitive displacement of calcium (and to a lesser extent zinc) from calcium-binding proteins, including calmodulin, protein kinase C (PKC), and N-methyl-D-aspartate (NMDA) receptors [12]. By activating PKC at concentrations several orders of magnitude lower than Ca²⁺, Pb permanently alters synaptic plasticity and glutamate neurotransmission, impairing learning and memory. In vivo rodent studies have demonstrated reduced spatial learning (Morris Water Maze performance), lowered hippocampal long-term potentiation (LTP), and reduced brain-derived neurotrophic factor (BDNF) mRNA at blood Pb levels as low as 5–10 μg/dL—concentrations achievable in occupationally exposed cosmetics workers [28].

Haematopoietically, Pb inhibits δ-aminolaevulinic acid dehydratase (ALAD) and ferrochelatase, blocking haem biosynthesis and causing protoporphyrin accumulation in erythrocytes. Clinically, this manifests as normocytic anaemia and elevated urinary δ-ALA, biomarkers detectable at BPb ≥ 10 μg/dL [12]. Cardiovascular effects are mediated through oxidative stress–induced endothelial nitric oxide synthase (eNOS) uncoupling, which reduces vascular NO bioavailability and promotes hypertension—consistent with the hypertension and cardiovascular disease burden observed in long-term dye users in this cohort [29]. Lead is also an epigenetic disruptor: in utero and early-life Pb exposure induces persistent DNA methylation changes (particularly hypomethylation of LINE-1 elements and hypermethylation of CDKN2A) that alter cancer susceptibility decades after exposure cessation, providing a plausible mechanism for the IARC Group 2B carcinogenicity classification [30].

#### 4.4.2 Manganese (Mn)

While essential at trace levels, Mn at the concentrations found in products HD-J, HD-C, and HD-I (155–295 ppm) poses substantial neurotoxic risk, particularly through the inhalation pathway when powdered dyes are mixed and applied in confined, poorly ventilated spaces [13]. Mn is preferentially transported across the blood–brain barrier by the divalent metal transporter 1 (DMT-1), accumulating in the globus pallidus, striatum, and substantia nigra pars reticulata—brain regions critical for motor control [13].

The primary in vivo mechanism involves mitochondrial dysfunction: Mn accumulates in mitochondria, inhibits Complexes I and III of the electron transport chain, and drives superoxide production. The resulting oxidative stress depletes glutathione and activates microglia, leading to neuroinflammatory cascades mediated by TNF-α, IL-6, and NF-κB signalling [31]. In vivo rodent studies with oral Mn at 10–50 mg/kg/day demonstrate progressive Parkinsonian motor deficits including tremor, rigidity, bradykinesia, and impaired righting reflex, accompanied by dopaminergic neuronal loss in the substantia nigra [31]. At the protein level, Mn accelerates the oligomerisation and aggregation of α-synuclein—the hallmark pathology of Parkinson’s disease—through a mechanism involving Mn²⁺ binding to the N-terminal domain of the protein [13]. The six-fold EDI elevation observed under the occupational versus consumer scenario is critical: at 200 g/day contact with HD-J, dermal and inhalation Mn intake approximates occupational exposure levels documented to cause manganism in welders and miners.

#### 4.4.3 Cadmium (Cd)

Cadmium is a toxicologically unique metal in that it is essentially non-essential, is excreted extremely slowly (biological half-life 10–30 years in the kidney), and induces its own transport/storage protein (metallothionein, MT) as part of a cellular defence that paradoxically becomes toxic when exhausted. At the kidney cortex, the principal target organ, Cd²⁺ transported as Cd-MT complexes undergoes lysosomal degradation, releasing free Cd²⁺ that overwhelms MT synthesis capacity, causing proximal tubular cell death—manifesting clinically as β₂-microglobulinuria, glucosuria, aminoaciduria, and the Fanconi syndrome of complete proximal tubular dysfunction [11].

Carcinogenicity involves multiple mechanisms. Cd²⁺ directly damages DNA through strand breaks and oxidised purine adducts while simultaneously inhibiting base-excision repair enzymes (OGG1, APE1), creating a state of unrepaired oxidative genomic damage [32]. Epigenetically, Cd induces global DNA hypomethylation and locus-specific hypermethylation of tumour suppressor gene promoters (e.g., MLH1, RASSF1A, E-cadherin), driving aberrant gene silencing independent of direct DNA mutation [32]. In vivo, subcutaneous Cd injection in rodents induces testicular tumours; chronic oral/inhalation exposure causes lung adenocarcinoma; and epidemiological cohorts show a 1.2–1.7-fold elevation in post-menopausal breast cancer risk per doubling of urinary Cd [11], [33]. Endocrine disruption occurs through Cd²⁺ binding to the oestrogen receptor α (ERα) ligand-binding domain, activating oestrogenic transcription at concentrations as low as 10⁻⁹ M—concentrations achievable from occupational skin absorption [34].

#### 4.4.4 Mercury (Hg)

Inorganic mercury (Hg²⁺), detected in HD-A, HD-E, and HD-H, exerts toxicity primarily through high-affinity binding to sulfhydryl (-SH) groups in enzymes and structural proteins, effectively blocking all thiol-dependent biological processes [35]. Key enzymatic targets include glutathione peroxidase (GPx), thioredoxin reductase, and the selenoenzymes—disrupting the intracellular antioxidant network and causing an oxidative stress milieu characterised by lipid peroxidation, mitochondrial membrane depolarisation, and apoptotic caspase activation [35]. In vivo, repeated low-dose Hg²⁺ exposure in rats causes cerebellar Purkinje cell loss, peripheral sensory neuropathy, and renal glomerular damage [35]. Critically, the gut microbiome converts a fraction of ingested inorganic Hg to methylmercury (MeHg), which crosses the BBB with high efficiency and accumulates in brain tissue at concentrations far exceeding blood levels, amplifying the apparent neurotoxic dose from skin-absorbed Hg that is partially excreted in bile and subsequently reabsorbed.

#### 4.4.5 Nickel (Ni)

Nickel is the most prevalent contact allergen in the Western world and a WHO- and IARC-recognised human carcinogen (Group 1) in occupational inhalation settings. Dermally, Ni²⁺ acts as a hapten: it binds to extracellular and intracellular proteins (notably histidine-rich peptides presented by HLA-DR), forming immunogenic Ni-protein conjugates recognised by allergen-specific T cells [14]. A single sensitisation event—which can occur at Ni concentrations as low as 5 mg/cm²/week—primes long-lived memory T cells that subsequently elicit allergic contact dermatitis (ACD) upon each re-exposure [14]. The Ni concentration in HD-C (6.10 ppm)—the highest detected value—is sufficient to elicit sensitisation in genetically predisposed individuals (HNMT*2 polymorphism carriers) even through the partial dermal exposure modelled here.

At the molecular level, Ni²⁺ inhibits the 2-oxoglutarate-dependent dioxygenases, including prolyl hydroxylases (PHDs) and jumonji-domain histone demethylases, inducing a pseudo-hypoxic transcriptional state (HIF-1α stabilisation) and generating activating H3K9me2 marks in regions associated with oncogene expression [36]. Ni also generates reactive oxygen species through Fenton-like reactions with H₂O₂, causing 8-OHdG adducts and DNA strand breaks. In vivo, intramuscular nickel subsulfide injection in rodents produces rhabdomyosarcoma, and occupational cohort studies demonstrate 1.5–2.5-fold increased lung and nasal cancer risk in refinery workers [36].

#### 4.4.6 Copper (Cu)

Although Cu is an essential micronutrient, Cu²⁺ at elevated concentrations shifts from antioxidant cofactor to pro-oxidant catalyst through Fenton-like redox cycling: Cu(II) + H₂O₂ → Cu(I) + ·OH + OH⁻. In vivo, chronic topical Cu²⁺ exposure generates OH · radicals that oxidise membrane lipids, cross-link collagen, and fragment DNA strands [37]. At the hair follicle, excessive Cu competes with Zn²⁺ for metallothionein-binding and disrupts Zn-dependent keratinocyte differentiation enzymes, providing a mechanistic explanation for the hair loss reported by 16.9% of hair dye users in this study. Elevated systemic Cu is associated with neurotoxicity in Wilson’s disease models (hepatic Cu overload triggering hippocampal neurodegeneration) and with accelerated amyloid-β oligomerisation in Alzheimer’s disease pathological models, though these represent extreme exposure scenarios beyond what dermal cosmetic use alone would produce.

### 4.5 Integration of biomonitoring, risk assessment, and symptom data

The three evidence streams, namely chemical analysis, pilot biomonitoring, and self-reported health effects, are mutually informative but should not be interpreted as proving causation. In the GFAAS subset, detectable blood Pb occurred only among participants with at least 20 years of occupational exposure. This pattern is biologically plausible given the long skeletal residence time of Pb, but the small, purposively selected sample cannot establish an exposure threshold or a population-level association. Larger studies with repeated measurements and adjustment for environmental and dietary sources are required [12].

Symptoms reported by participants with detectable Pb, including sinusitis, blurred vision, eye redness, and hypertension, are compatible with several occupational and environmental exposures but are not specific to heavy metals. Organic dye constituents, workplace aerosols, diet, water, traffic emissions, and other background sources may also contribute. Accordingly, the symptom findings provide context and biological plausibility but do not demonstrate that hair-dye metals caused the reported conditions

### 4.6 Regulatory and public health implications

The unacceptable Cd carcinogenic risk for occupational workers (LCR 4.37 ×10⁻⁴ from HD-D) demands immediate regulatory response. We recommend that Sudan’s National Drug and Cosmetics Authority (NDCA): (i) mandate pre-market heavy metal screening (FAAS or ICP-MS) of all imported hair dye products, with specific attention to powdered formulations from Asian origins; (ii) adopt the EU technically-avoidable limits as interim maximum concentrations (Pb ≤ 2 ppm; Cd ≤ 0.1 ppm; Hg ≤ 0.1 ppm); (iii) establish a biomonitoring programme with annual blood Pb screening for beauty workers and Cd biomarkers (urinary β₂-microglobulin and Cd) for those with ≥ 10 years of occupational exposure; and (iv) require PPE provision and training as a condition of business licensing for hair salons.

At the product level, the clear gradient between European cream formulations (no detectable metals) and Asian powdered formulations (highest metal burden) supports differentiated regulatory stringency: powdered and stone-based dyes should be subject to enhanced scrutiny and potentially restricted pending testing. Consumer education should target the specific risk of cheaper, darker powdered formulations and the importance of PPE and workspace ventilation even for personal use.

### 4.7 Limitations

Several limitations qualify these findings. The biomonitoring component was a small pilot group (n = 19) recruited purposively, which limits generalisability, increases the possibility of selection bias, and precludes robust multivariable correlation analysis. The cross-sectional design prevents causal inference. Questionnaire symptoms and product types were self-reported, multiple-product use was possible, and product-specific symptom associations were therefore not estimated. Environmental Pb sources, including water, food, air, soil, traffic, and prior exposures, were not measured; consequently, the proportion of blood Pb attributable specifically to hair dyes cannot be determined. Risk estimates were based on fixed parameters. The selected DAF values were obtained from published experimental or regulatory sources rather than measured in the tested formulations, and true absorption may vary with speciation, vehicle, skin integrity, contact time, and occlusion. Oral RfDs and CSFs were used as screening surrogates where dermal toxicity values were unavailable, which adds uncertainty and may overestimate or underestimate risk. A simple proportional sensitivity interpretation indicates that halving or doubling a DAF would halve or double the corresponding EDI, HQ, and LCR; therefore, the numerical estimates should be interpreted as screening-level signals. Multi-route exposure, particularly inhalation during powder mixing, was not quantified.

## 5 Conclusions

This study provides integrated screening-level evidence on heavy metals in selected hair dyes sold in Khartoum, together with exploratory biomonitoring and model-based dermal risk estimates. Manganese and lead were the most prominent contaminants among the tested products, while concentrations varied substantially across formulations. Because the product and biomonitoring samples were limited, the findings should not be generalised to all hair dyes or exposed women in Sudan.

Under the stated assumptions, individual-product non-carcinogenic hazard indices remained below 1, although occupational estimates for high-lead products approached this threshold. Cadmium in HD-D produced the strongest modelled cancer-risk signal, exceeding 10^-6 for consumers and 10^-4 for occupational workers. These values are screening estimates because formulation-specific dermal absorption was not measured and oral toxicity values were used as surrogates where dermal values were unavailable.

Established toxicological evidence supports the biological plausibility of neurological, renal, cardiovascular, and dermatological effects from excessive exposure to the detected metals. However, the present cross-sectional data do not demonstrate that the participants' self-reported symptoms were caused by hair-dye metals.

The results support strengthened pre-market testing of cosmetic dyes, improved workplace ventilation and personal protective equipment, and larger biomonitoring studies that measure relevant environmental and occupational co-exposures. Priority should be given to products and work practices associated with frequent handling of powdered or stone-based dyes.

## CRediT authorship contribution statement

**Ahmed H. Bakheit:** Writing – review & editing, Methodology, Investigation, Formal analysis, Conceptualization. **Ragaa Satti Mohmmed Abadi:** Writing – review & editing, Validation, Methodology, Data curation. **Raja Y. Alghadi:** Writing – review & editing, Validation, Resources, Methodology, Formal analysis. **Badreddin M.H. Al-Hadiya:** Writing – review & editing, Validation, Supervision, Conceptualization. **Fatima Abdalaziz Hassan Mohamed:** Writing – original draft, Methodology, Investigation, Formal analysis, Data curation, Conceptualization.

## Declaration

During the preparation of this work, the author(s) used AI-based tools (Microsoft Copilot and Claude) for language editing and stylistic improvements. The author(s) reviewed and edited the generated content as necessary and take full responsibility for the final published version.

## Funding

This research received no specific external funding.

## Declaration of Competing Interest

The authors declare that they have no known competing financial interests or personal relationships that could have influenced the work reported in this manuscript.

This research received no specific grant from any funding agency in the public, commercial, or not-for-profit sectors. All authors have reviewed and approved the final version of the manuscript and declare no conflicts of interest related to the research, authorship, or publication of this work.

## Data Availability

Data will be made available on request.
